# Supplementary material for: Integration of Mechanical Testing, In Vivo Optical Coherence Elastography and Personalized Finite Element Modeling to Predict Geometrical Outcomes of Corneal Cross-Linking
Source: Ann Biomed Eng. 2026 Jun 16;54(8):2786–800. doi: 10.1007/s10439-026-04236-0 (PMC13391455; doi:10.1007/s10439-026-04236-0)
Supplement: Supplementary file 1 — Supplementary file1 (PDF 1200 KB) [file 10439_2026_4236_MOESM1_ESM.docx]

Supplementary Material

**Integration of mechanical testing, *in vivo* optical coherence elastography and personalized finite element modeling to predict geometrical outcomes of corneal cross-linking**

**Authors:** Matteo Frigelli^1^, Robert Lohmüller^2^, Miguel A. Ariza Gracia^1^, Günther Schlunck^2^, Stefan J. Lang^3^, Emilio A. Torres-Netto^4^, Farhad Hafezi^4,5,6^, Philippe Büchler^1^, Sabine Kling^1^

^1^ ARTORG Center for Biomedical Engineering Research, University of Bern, Bern, Switzerland

^2^ Eye Center, Faculty of Medicine, University of Freiburg, Freiburg, Deutschland

^3^ Department of Ophthalmology, University Hospital Brandenburg, Brandenburg an der Havel, Germany

^4^ ELZA Institute AG, Zürich, Switzerland

^5^ Faculty of Medicine, University of Geneva, Geneva, Switzerland

^6^ Department of Ophthalmology at New York University Grossman School of Medicine, New York University, New York, USA

**Corresponding Author:**

matteo.frigelli@unibe.ch

# Appendix A – Nanoindentation

In the NI experiments, the Hertz’s modulus $E_{HZ}$ was obtained by fitting the following equation to the force-depth curve in the region encompassed between 10% and 98% of the maximum load:

|  | $F=$ $\frac{4}{3}E\sqrt{R}{(h)}^{\frac{3}{2}}$ | (A.1) |
| --- | --- | --- |

*F* represents the indentation force, *R* the radius of the indenter tip (0.5 mm), and *h* the indentation depth.

The indentation creep $C_{IT}$ was calculated according to the standard ISO 14577:

|  | $C_{IT}=100*$ $\frac{h_{m}-h_{i}}{h_{m}}$ | (A.2) |
| --- | --- | --- |

where the indentation depths at the beginning and at the end of the hold period are given by $h_{i}$ and $h_{m}$, respectively.

The locations within the central cornea at which NI measurements were conducted are indicated by black dots in Figure A1.


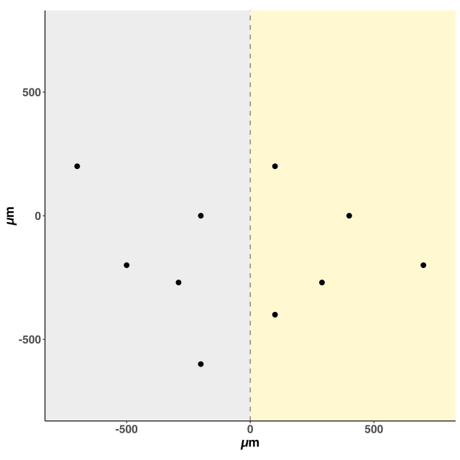


**Figure A1:** regions of the cornea (1 mm diameter area) where NI was performed (n = 10 indentations per sample, indicated by black dots). The yellow region denotes the area treated with CXL.

# Appendix B – Optical Coherence Elastography

The algorithm for the OCE strain computation is detailed herein. Briefly, a phase-sensitive deformation tracking algorithm was used to obtain the phase difference between the corresponding A-scans in the reference and deformed (Δp=2 mmHg) configurations by amplitude-weighted complex cross-correlation:

|  | $C(z, x)=\sum_{j=-v_{z}}^{v_{z}} \sum_{k=-v_{x}}^{v_{x}} A(z+j, x+k) \cdot A_{\Delta p}^{*}(z+j, x+k)$ | (B.1) |
| --- | --- | --- |

Where A(z, x) is the complex OCT interference signal acquired at position (z,x) [m] and v_z_ = 3 and v_x_ = 3 [pixels] is the size of the phase-processing windows used. The phase difference was employed to obtain the pixel-wise strain in the direction of the optical axis, $\varepsilon_{zz}$ [-] by deriving the axial displacement U(z,x) [m] along z:

|  | $\varepsilon_{zz}\left( z,x \right)= \frac{dU}{dz}=\frac{d}{dz}\left( \frac{\lambda_{mean}*\angle C\left( z,x \right)}{4\pi n} \right)= \frac{\lambda_{mean}*\angle R\left( z,x \right)}{4\pi n\delta}$ | (B.2) |
| --- | --- | --- |

Where n = 1.375 [-] is the refractive index of the cornea, δ = 4.48 μm is the OCT axial sampling unit (in air), ∠R is the angle of a second complex cross-correlation $R\left( z, x,y \right)=\sum_{j=-w_{z}}^{w_{z}} \sum_{k=-w_{x}}^{w_{x}} W\left( z+j,x+k \right) \cdot W^{*}\left( z+1+j, x+k \right)$ and w_z_ = 3 and w_x_ = 3 [pixels] is the size of the phase processing windows used. Inevitably, the application of phase-processing windows in both cross-correlations resulted in the axial and lateral resolutions of the strain maps being reduced to 39 and 144 μm, respectively, which is lower than the original resolution of the structural images.

# Appendix C – Hyperelastic Material Model

To exhaustively describe the hyperelastic material model we reported in the method section, we have to start from eq. (1) of this manuscript. $\bar{I}_{1}$ and $\bar{I}_{4}$ are invariants of the isochoric Cauchy-Green strain tensor. $\bar{C}=\boldsymbol{J}^{\boldsymbol{2/3}}\boldsymbol{F}^{\boldsymbol{T}}\boldsymbol{F}$, $\boldsymbol{F}$ is the deformation gradient tensor, and $J=det(\boldsymbol{F)}$. The Macaulay bracket operator, denoted as < • >, is employed to account for the fact that collagen fibers only contribute to the overall mechanical response when they are in a state of tension:

|  | $\begin{aligned} &\bar{I}_{1}=\mathrm{Tr}\bar{C} \\ &\bar{I}_{4}=(\mathbf{a}\otimes\mathbf{a}):\bar{C} \\ &\langle\bar{E}\rangle=\left\{ \begin{matrix} \bar{E} & \text{ if }\bar{E}>0 \\ 0 & \text{ if }\bar{E}\leq0 \end{matrix} \right. \end{aligned}$ | (C.1) |
| --- | --- | --- |

The general fiber direction in spherical coordinates, 𝒂, is described as:

|  | $\begin{aligned} \boldsymbol{a (}\varphi,\theta\boldsymbol{)}=cos\varphi sin\theta\boldsymbol{e}_{\boldsymbol{1}}+sin\varphi sin\theta\boldsymbol{e}_{\boldsymbol{2}}+cos\theta\boldsymbol{e}_{\boldsymbol{3}} \\ 0 \leq\phi\leq2\pi, 0 \leq\theta\leq\pi\end{aligned}$ | (C.2) |
| --- | --- | --- |

The angular density of the fiber distribution ρ was decomposed as a product of the in-plane and out-of-plane distributions $\rho_{in}$ and $\rho_{out}$:

|  | $\begin{aligned} \rho(\varphi,\theta)&=\rho_{op}(\theta)\rho_{ip}(\varphi) \\ \rho_{op}(\theta)&=2\sqrt{\frac{2b}{\pi}}\frac{\exp\left[ -2b\cos^{2}\theta\right]}{\mathrm{erf}(\sqrt{2b})},0\leq\theta\leq\pi\\ \rho_{ip}(\varphi)&=\frac{\exp[a\cos(2\varphi)]}{I_{0}(a)},0\leq\varphi\leq2\pi\end{aligned}$ | (C.3) |
| --- | --- | --- |

Where, $I_{0}$ is the modified Bessel function of the first kind of order 0. The fiber distribution parameters $a$ and $b$ were defined based on x-ray scattering and second harmonic generation data. While $b$=2.5 was kept constant along the corneal curvature, the parameter $a$ went from $a$=0 (isotropic in-plane dispersion) in the anterior part to $a$=5 (aligned fibers direction) in the posterior part of the cornea.

**Table C1:** Material parameters adopted in the FEM for both the pre- and post- CXL models.

| $\boldsymbol{k}$  **[MPa]** | $\boldsymbol{C}_{\boldsymbol{10}}$  **[MPa]** | $\boldsymbol{k}_{\boldsymbol{1}}^{\boldsymbol{ant}}$  **[MPa]** | $\boldsymbol{k}_{\boldsymbol{2}}$  **[-]** | $\boldsymbol{f}_{\boldsymbol{c}}$  **[-]** | $\boldsymbol{a}_{\boldsymbol{ant}}$  **[-]** | $\boldsymbol{a}_{\boldsymbol{post}}$  **[-]** | $\boldsymbol{b}$  **[-]** |
| --- | --- | --- | --- | --- | --- | --- | --- |
| 10^3^ | 0.025 | 1.45 | 18.9 | 0.02 | 0 | 5 | 2.5 |

# Appendix D – Corneal Meshing Algorithms

This study employed two different meshing approaches to create a FEM of the cornea (Figure D1). For identifying *ex vivo* mechanical parameters, an average cornea model was used to reduce computational costs, assuming corneal geometry has minimal effect on NI experiments. When applied to the *in vivo* clinical setting, however, a patient-specific approach was adopted to generate meshes tailored to individual corneal geometries, ensuring accurate curvature analysis in post-CXL evaluation.


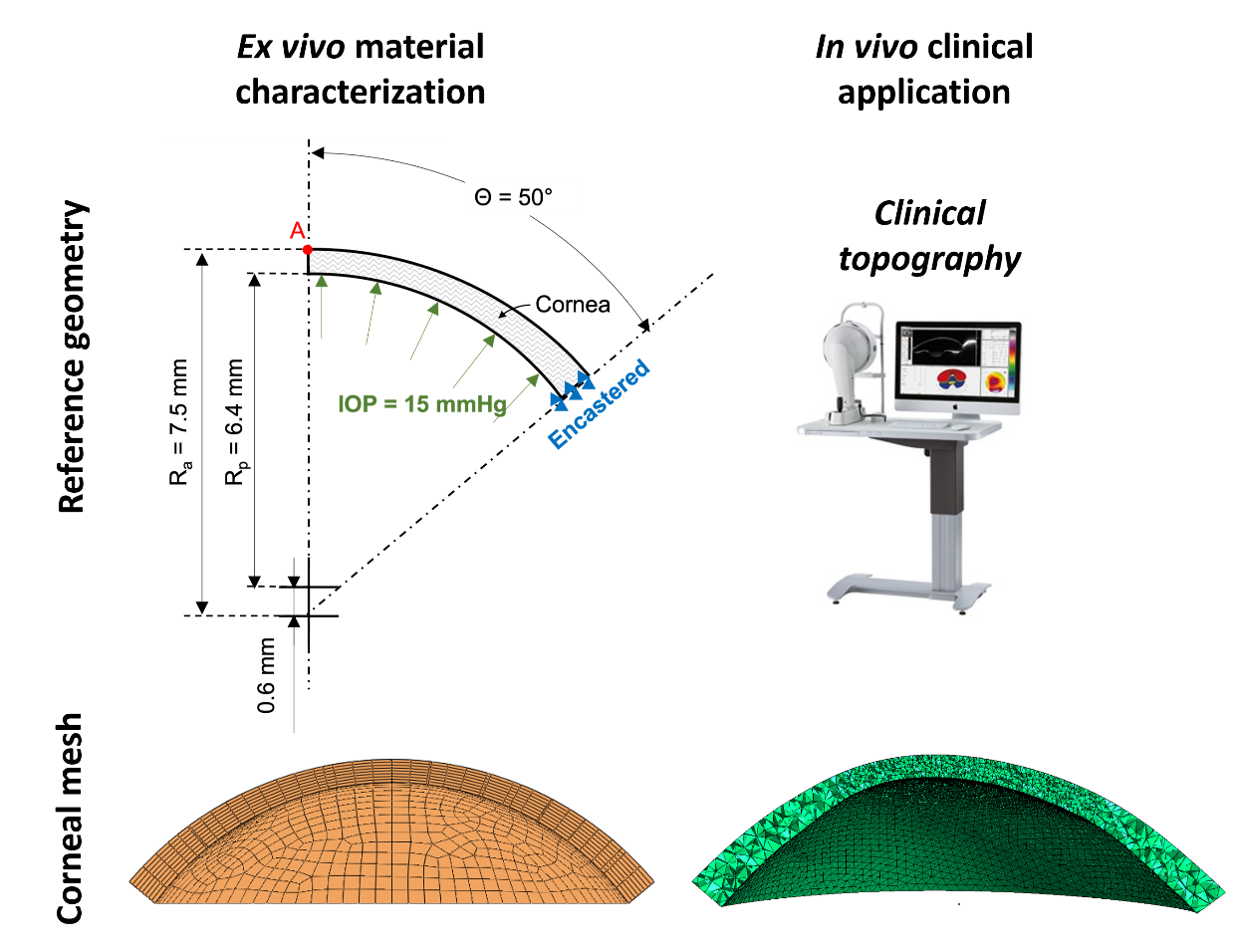


**Figure D1:** different corneal reference geometries and corresponding meshes adopted in the different phases of the present study.

# Appendix E – Supplementary Experimental Analyses

## Methods

### Uniaxial tensile test

Strips measuring 6x2 mm were cut from both the CXL-treated and non-CXL portions of the cornea using the Femto LDV Z8 Neo femtosecond laser (Ziemer Ophthalmic Systems AG, Switzerland). These strips were then preserved overnight in a MEM 15% dextran solution. Each strip was subjected to uniaxial testing using the UStretch device (CellScale, Waterloo, Canada). The tests were conducted at room temperature with the strips maintained in the culture medium bath. Each strip was pre-stretched with a force of 10 mN and subjected to 6 loading cycles at a strain rate of 0.16%/s, each reaching a strain of 10%. The force-displacement data from the fifth cycle were analyzed, and the tangential elastic modulus at 10% strain ($E_{10}$) was considered for comparing the stiffness of the UV-irradiated regions vs. the non-irradiated ones.

## Results

### Uniaxial tensile test

Figure E1 shows that the corneal tissue exhibited a stiffer, but statistically non-significant behavior under UTT after CXL, which was reflected by an increased tangent modulus (124±77 kPa vs 164±70 kPa; p=0.125). Out of n=5 corneas considered, one was discarded since one of the strips got damaged during the mounting phase.


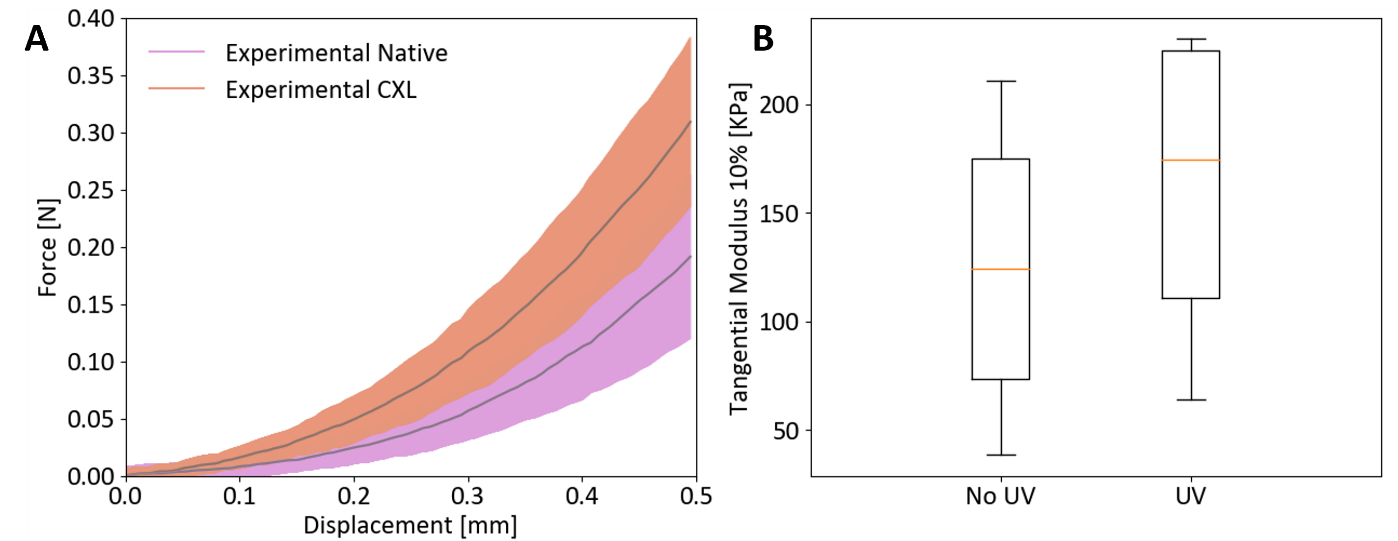


**Figure E1:** no-UV vs UV region UTT tensile test results (n=4). A) experimental force displacement curves. B) tangential modulus at 10% strain distributions.

### Nanoindentation

As shown in Figure E2, the non-irradiated region reported a slightly higher $E_{HZ}$ when compared to the irradiated one after CXL, with values ranging from 145.7±7.5 kPa to 140.9±4.7 kPa (p<0.01), respectively. The decrease in $E_{HZ}$ here reported (4.75±9.6 kPa) is in the range of the standard deviations of the NI measurements performed in this work. No statistically significant variations in $C_{IT}$ were reported between these two areas of the cornea (16.23±1.4 % vs 16.51±1.3 %, p=0.09, for non-UV and UV regions, respectively).


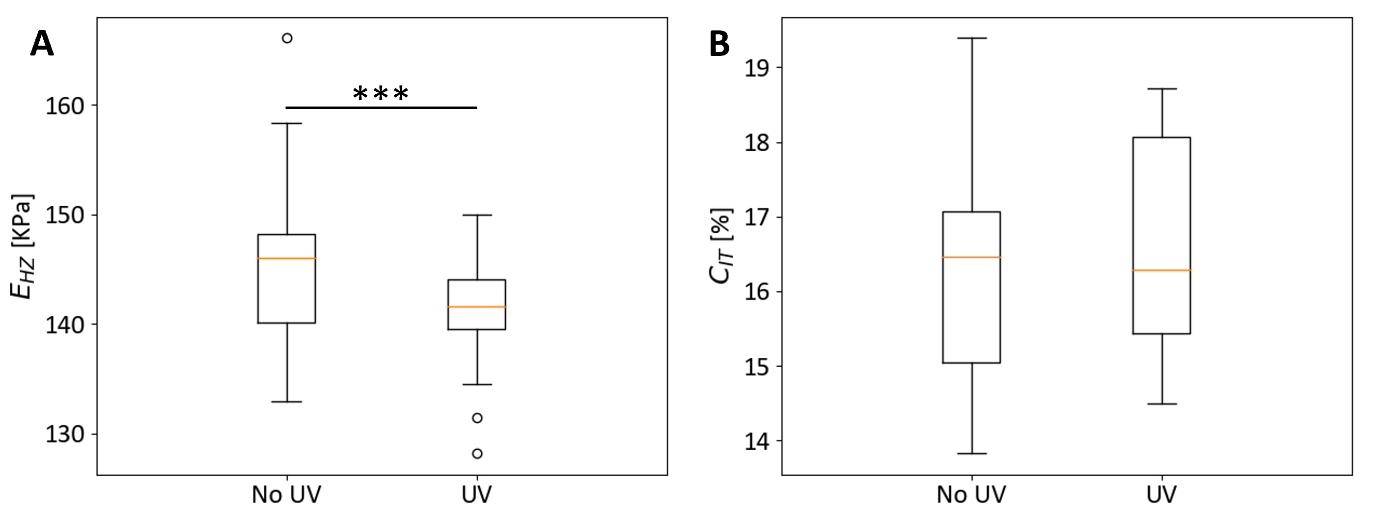


**Figure E2:** no-UV vs UV region NI test results (n=25). A) Elastic Hertz modulus $E_{HZ}$. B) indentation creep $C_{IT}$. ***.001≤p≤.01.

### Ex vivo optical coherence elastography

No statistically significant differences were observed between the non-irradiated and the irradiated regions in terms of $\varepsilon_{zz}$ measured via *ex vivo* OCE (0.5±3.1 ‰ vs 1.1±2.6 ‰, p=0.125, for the no-UV and UV regions, respectively), as reported in Figure E3. From the enface view in Figure E4 the patterned CXL effect of the FEM can be appreciated.


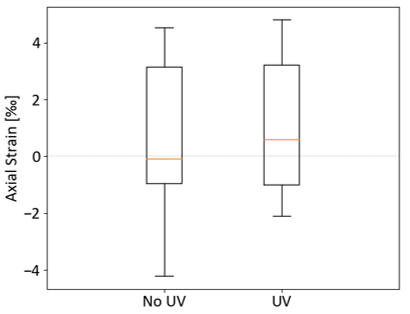


**Figure E3:** no-UV vs UV region *ex vivo* OCE inflation test results (n=5).


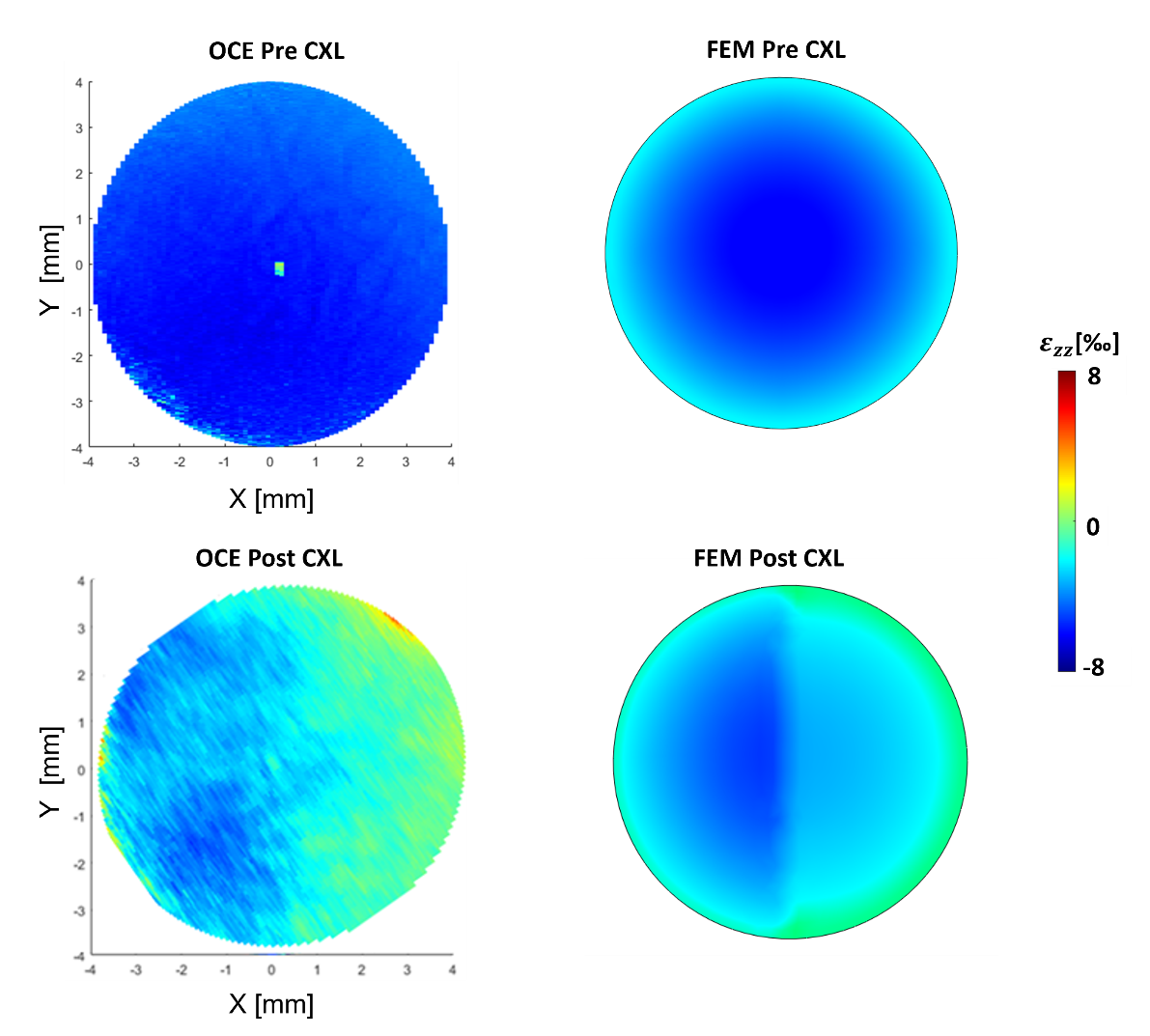


**Figure E4:** enface views for both OCE imaging (left column) and FE results (right column). The first row shows the pre-CXL condition, while the second row shows the post-CXL condition. Color scale represents axial strain $\varepsilon_{zz}$ [‰] values.
